# Bone turnover markers in serum but not in saliva correlate with bone mineral density

**DOI:** 10.1038/s41598-020-68442-z

**Published:** 2020-07-14

**Authors:** Katharina Kerschan-Schindl, Ewald Boschitsch, Rodrig Marculescu, Reinhard Gruber, Peter Pietschmann

**Affiliations:** 1 Department of Physical Medicine, Rehabilitation and Occupational Therapy, Medical University of Vienna, Vienna, Austria; 2 Klimax, Ambulatorium für Klimakterium und Osteoporose, Vienna, Austria; 3 Department of Laboratory Medicine, Medical University of Vienna, Vienna, Austria; 4 Department of Oral Biology, School of Dentistry, Medical University of Vienna, Vienna, Austria; 5 Department of Periodontology, University of Bern, Bern, Switzerland; 6 Institute of Pathophysiology and Allergy Research, Center for Pathophysiology, Infectiology and Immunology, Medical University of Vienna, Vienna, Austria

**Keywords:** Endocrine system and metabolic diseases, Metabolic bone disease, Osteoporosis, Prognostic markers, Oral medicine, Dental public health

## Abstract

Saliva was proposed as a diagnostic tool for systemic diseases. Here we determined the correlation of bone turnover markers in saliva, bone turnover markers in serum and bone mineral density in postmenopausal osteoporotic and healthy women. Forty postmenopausal osteoporotic and 40 age-matched healthy non-osteoporotic females were recruited for this case–control study. Serum and stimulated saliva levels of osteocalcin, N-terminal propeptide of type I collagen, bone-specific alkaline phosphatase and cross-linked-C-telopeptide of type I collagen were determined. Bone mineral density of the lumbar spine, proximal femur, and total hip were obtained. We show that osteocalcin and cross-linked-C-telopeptide of type I collagen (CTX) reached detectable levels in saliva while N-terminal propeptide of type I collagen and alkaline phosphatase were close or below the detection limit. Serum levels of bone turnover markers were significantly higher than saliva levels. Correlation analysis revealed a strong correlation of serum osteocalcin and, to a lesser extent, also serum CTX values with bone mineral density in lumbar spine, femoral neck, or total hip, respectively. There was, however, no significant correlation of bone mineral density with the respective bone turnover markers in saliva. There was a trend that saliva osteocalcin correlates with femoral neck (*p* = 0.16) or total hip (*p* = 0.06). There was also no association between serum and saliva bone turnover markers. This study reveals that saliva cannot replace the withdrawal of serum to evaluate bone metabolism.

## Introduction

Osteoporosis is defined as a skeletal disorder characterized by compromised bone strength and predisposing a person to an increased risk of fracture^1^. The consequences of fractures are pain, morbidity and an increased risk of mortality^2^. Particularly hip fractures are associated with a high socio-economic burden^2^. Despite the fact that effective diagnostic and pharmacological strategies to prevent osteoporotic fractures are available^3^, an enormous proportion of the patients is not diagnosed and not treated. Most patients with vertebral fractures did not receive dual energy X-ray absorptiometry scans within the preceding 2 years and did consequently not receive pharmacological therapy^4^. Hence, there is a need for simple screening tools to identify patients at risk of fractures.

Bone turnover markers cannot predict fractures but are used to assess the response to anabolic and antiresorptive therapies^5^. Among such markers osteocalcin (OC) and N-terminal propeptide of type I procollagen (P1NP) reflect bone formation while C-telopeptide of type I collagen (CTX) reflects bone resorption^5,6^. The retrieval of serum requires clinical indication and trained personnel. In addition, the analysis of bone remodeling markers in urine is uncommon without the analysis of serum parameters^5^. The necessary venipuncture is not only unpleasant but also technically demanding. The measurement of bone turnover markers in serum is therefore not ideal for screening. Determining bone turnover markers in saliva would be a potential noninvasive alternative to established serum analysis. The question arises as to whether there is a correlation between bone turnover markers in saliva and serum.

The clinical studies available so far do not provide a clear picture. Bullon et al. recruited 73 postmenopausal women collecting saliva from the sublingual zone with paper strips that were eluted in extraction buffer. Osteocalcin measurements were based on electrochemiluminescence technique. There was no difference in the concentration of osteocalcin in serum or saliva in osteoporotic patients compared to patients with normal bone density^7^. However, there was a tendency that serum rather than saliva osteocalcin is linked to bone mineral density^7^. The concentration of osteocalcin in serum or saliva was similar in the ng/ml range. Based on data from 37 women, McGehee et al.^8^ found a high correlation between salivary osteocalcin and bone mineral density. They collected stimulated saliva after jawing on paraffin and measured osteocalcin based on enzyme-linked immunosorbent assay^8^. No serum data are available. Pellegrini et al.^9^ report serum and salivary CTX levels but not related to bone mineral density. CTX levels were greatly lower in unstimulated saliva compared to serum^9^. Thus, there are few and controversial data on the use of saliva to analyze bone turnover markers.

Preclinical studies are also available. Johnson et al.^10^ demonstrated on ovariectomized sheep that osteocalcin in saliva correlates with bone density measured by contact radiography of excised hemimandibles. Salivary and serum osteocalcin concentrations increased twice within 4–8 months after ovariectomy. Osteocalcin levels were determined from serum by enzyme-linked immunosorbent assay and concentrations expressed as ng/ml. Saliva was collected using the passive drool collection method^10^. Pellegrini et al.^11^ showed an association of CTX in serum and saliva using ovariectomized and sham-operated rats. Salivation was stimulated by intraperitoneal injection of pilocarpine. Radiographic scanning was performed under light anesthesia. It is therefore particularly the preclinical data, emphasizing the potential of saliva to determine bone turnover markers. However, the data available clearly demonstrate the need for further clinical studies that investigate the association of bone turnover markers in saliva and serum, and analyze the possible relationships of bone turnover markers in saliva with bone density.

The overall aim of the present study was to extend on existing research and evaluate the possible use of stimulated saliva to serve as a potential source of bone turnover markers that correlate with serum makers and bone mineral density in a clinically relevant scenario. Considering that the geriatric population being at risk of osteoporosis and other metabolic bone diseases potentially suffers from xerostomia^12^, unstimulated saliva collection is not appropriate or convenient. Xerostomia, the subjective feeling of dry mouth is a symptom most frequently associated with alterations in the quality and quantity of saliva, affects at least one fifth in a European population^13,14^. To which extent, however, the blood–saliva barrier affects the accumulation of bone turnover markers in stimulated saliva has not been revealed. The majority of salivary proteins are secreted by salivary glands, while a comparatively low percentage of proteins derive from capillary leakage, thus directly from the blood stream^15^. In vitro models and modeling biomarker kinetics through the blood–saliva barrier has received increasing attention but the lack of a clear-cut understanding for dynamic passage of biomarkers from blood into the saliva remains an obstacle^15,16^. While keeping these limitations in mind, we proposed a clinically feasible approach to analyze bone turnover markers in stimulated saliva and correlate those with levels in serum and with bone mineral density.

## Methods

### Study design and study population

This was a single center, case–control, cross-sectional study including patients presenting at the menopause and osteoporosis outpatient clinic Klimax in Vienna, Austria, between October 2016 and September 2017. We enrolled 40 osteoporotic women and 40 controls. Included subjects were postmenopausal women between 50 and 80 years of age. Osteoporotic participants had to have primary osteoporosis (T-score at or below − 2.5 SD measured at the lumbar spine, femoral neck, or total hip) or osteopenia with a fragility fracture. To be eligible, participants were not allowed to be on drugs with potential effects on bone mineral density (BMD), like glucocorticoids, lithium, hormone—replacement therapy, selective estrogen-receptor modulators or oral bisphosphonates within the last 3 months and denosumab or parenteral bisphosphonates within the previous year. Exclusion criteria were fragility fractures within the past 6 months, malignant diseases within the previous 5 years, immobilization, renal or liver insufficiency, rheumatoid arthritis, or non-osteoporotic bone disease (for instance primary hyperparathyroidism or osteomalacia). The control group consisted of age-matched postmenopausal women with osteopenia (measured at the lumbar spine, femoral neck or, total hip) but no fragility fractures or with normal BMD. Exclusion criteria were the same as for the osteoporosis group.

### Study procedures

In all subjects, medical history was obtained and physical examination was performed. Prior to the study examinations, anthropometric measures were performed. Standing height was measured in stocking feet to the nearest centimeter using a stadiometer, and weight was measured using a balance beam scale, recalibrated monthly. A venous blood sample for the determination of routine parameters and bone turnover markers was drawn and saliva was collected in a standardized manner by 5 min chewing on a piece of paraffin (Ivoclar Vivadent AG, Schaan, Liechtenstein)^9^. After centrifugation serum and saliva samples were stored at − 70 °C until analysis. BMD was measured at the lumbar spine and hip region. The study protocol was approved by the ethics committee of the Medical University of Vienna (1511/2016). All subjects gave written informed consent.

### Biochemistry

The following BTMs were evaluated in serum and saliva by electrochemiluminescence immunoassays (ECLIA) on Cobas e 602 immunology analyzers (Roche Diagnostics, Switzerland): osteocalcin (OC; detection limit: 0.5 ng/ml; intra-assay coefficient of variation: 0.9–1.3%. inter-assay coefficient of variation: 1.2–2.3%), N-terminal pro-peptide of type I collagen (P1NP; detection limit: 5 ng/ml; intra-assay coefficient of variation: 1.6–3.5%; inter-assay coefficient of variation: 2.0–3.8%), and cross-linked-C-telopeptide of type I collagen (CTX; detection limit: 0.01 ng/ml; intra-assay coefficient of variation: 1.2–4.7%. inter-assay coefficient of variation: 1.5–5.7%). Bone alkaline phosphatase was measured by Liaison BAP Ostase automated CLIA on a Liaison XL analyzer (DiaSorin, Italy) with a specified detection limit of 0.1 ng/ml. All biochemical analyses were performed in accordance with the relevant guidelines and regulations in ISO 9001 certified and ISO 15189 accredited laboratories at the Department of Laboratory Medicine, Medical University of Vienna. For the bone turnover markers which we could detect reliably in saliva, OC and CTX, recovery and linearity were proven by spiking saliva samples with the respective high concentration controls provided by the manufacturer (PC Varia 2, OC 95.5 ng/ml, CTX 0.81 ng/ml) in various proportions. Intraindividual biological variability in saliva was estimated by analyzing samples collected on three consecutive days from three individuals.

### Bone mineral density measurement

Bone mineral density (BMD) measures of the lumbar spine and the hip region were performed by dual energy X-ray absorptiometry (DXA) using one single device for all patients (Prodigy fan beam; Lunar series, General Electrics Healthcare, Munich, Germany). All measurements were conducted using the standard procedures recommended by the manufacturer and the device was calibrated and a spine phantom was scanned to monitor performance every day.

### Statistical analysis

Data from the postmenopausal osteoporotic women and the age-matched healthy non-osteoporotic females were compared with the Mann–Whitney test. A Spearman’s rank correlation coefficient was calculated for every pair of data sets presenting the r and the *p* values. Statistical analyses were done using Prism 7 for Mac (GraphPad Software).

## Results

### Patient characteristics

**Figure 1:**
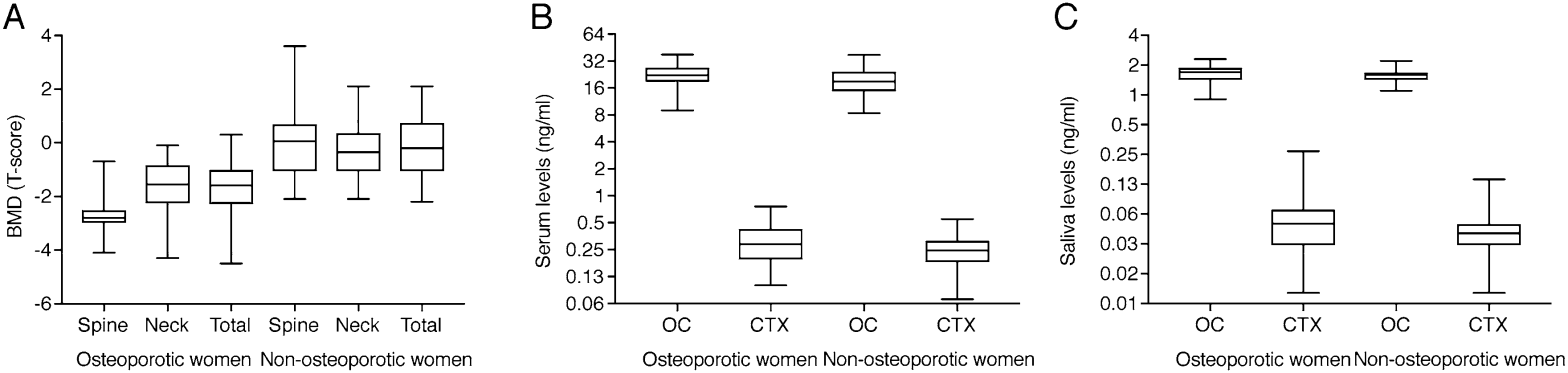
Overview of bone mineral density and bone turnover markers in serum and saliva. The data represent 40 postmenopausal osteoporotic women and 40 healthy non-osteoporotic females with bone mineral density (BMD) measured in the lumbar spine (Spine), femoral neck (Neck), or total hip (Total) expressed as T-score (**A**) and bone turnover markers osteocalcin (OC ng/ml) and cross-linked-C-telopeptide of type I collagen (CTX ng/ml) measured in serum (**B**) and saliva (**C**).

The data represent postmenopausal osteoporotic women with a median age of 64.5 years (min 59.3, max 70.0) and a body mass index of 24.0 (min 20.9, max 26.4) and healthy non-osteoporotic females with median age of 64.5 years (min 59.3, max 70.0) and body mass index of 27.9 (min 22.7, max 29.8) (Table 1). As indicated in Fig. 1A, osteoporotic women compared to non-osteoporotic females had the expected (*p* < 0.0001) lowered BMD of the lumbar spine (− 2.60 ± 0.72 vs − 0.04 ± 1.34), the femoral neck (− 1.63 ± 1.02 vs − 0.28 ± 1.05) and the total hip (− 1.67 ± 1.00 vs − 0.15 ± 1.17).

**Table 1:** Characteristics of the studied groups.

Variable	Osteoporotic patients	Healthy controls	*p* value
Age (a)	64.5 [59.3;70.0]	63.5 [56.3;71.0]	n.s
BMI	24.0 [20.9;26.4]	27.9 [22.7;29.8]	0.012
Fractures peripheral (n)	10	4	n.s
Fractures spine (n)	10	2	n.s
Smokers (n)	5	4	n.s
Periodontitis (n)	3	7	n.s
Ca supplementation (n)	24	7	0.001
Vitamin D supplementation (n)	34	20	0.007
Creatinin [mg/dl]	0.75 [0.70;0.83]	0.79 [0.70;0.80]	n.s
eGFR [mg/dl]	83.9 [73.0;91.3]	88.0 [75.8;93.5]	n.s
TSH [U/ml]	1.48 [1.01;2.25]	1.53 [1.26;2.06]	n.s
fT4 [pmol/l]	10.6 [9.6;12.9]	11.1 [9.3;12.3]	n.s
GGT [U/l]	15.0 [12.0;20.0]	21.5 [14.5;30.0]	0.01
C-reactive protein [mg/dl]	0.15 [0.07;0.37]	0.20 [0.06;0.39]	n.s
Vitamin D [ng/ml]	28.5 [21.8;39.0]	28.0 [21.0;33.7]	n.s

*eGFR* estimated glomerular filtration rate, *TSH* thyroid-stimulating hormone, *fT4* free thyroxin, *GGT* gamma-glutamyltransferase.

### Measurement of bone turnover markers in saliva

Bone specific alkaline phosphatase could not be detected at all and P1NP only in 22 of 76 of the saliva samples (29%). These parameters were therefore not further used. For OC and CTX, serial spiking experiments showed good recovery and linearity over the relevant concentration ranges: C_Expected_ = 0.889 * C_Measured_ − 0.789, R^2^ = 0.983 for OC and C_Expected_ = 1.211 * C_Measured_ − 0.017, R^2^ = 0.997 for CTX, respectively. The variation coefficients of saliva samples collected on consecutive days were 1.9–6% for OC and 13.3–20.1% for CTX.

### Bone turnover markers in serum and saliva in osteoporotic and healthy women

An overview of osteocalcin and CTX in serum and saliva in postmenopausal osteoporotic women and healthy non-osteoporotic females is presented in Fig. 1B, C, respectively. Comparing serum osteocalcin (22.73 ± 7.39 vs 19.73 ± 6.77; *p* = 0.059) and CTX (0.32 ± 0.16 vs 0.26 ± 0.12; *p* = 0.057) in postmenopausal osteoporotic women with those in healthy non-osteoporotic females failed to reach the level of significance. Also, saliva bone turnover markers in postmenopausal osteoporotic women compared to healthy non-osteoporotic females, showed no difference for OC (1.63 ± 0.35 vs 1.58 ± 0.25; *p* = 0.329) and CTX (0.060 ± 0.049 vs 0.047 ± 0.024; *p* = 0.315).

### Serum but not saliva bone turnover markers correlate with bone mineral density

**Figure 2:**
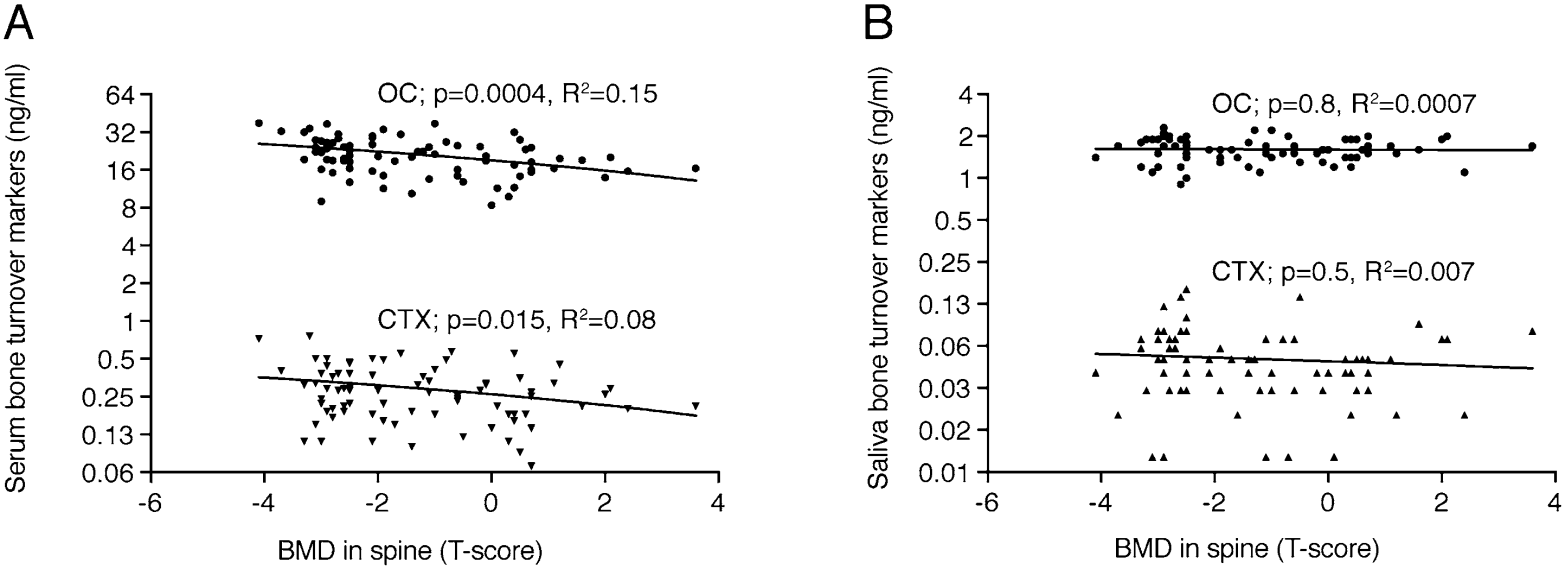
Correlations of bone turnover markers determined in saliva and serum with bone mineral density. The data represent 40 postmenopausal osteoporotic women and 40 healthy non-osteoporotic females with bone mineral density (BMD) measured in the lumbar spine expressed as T-score and bone turnover markers osteocalcin (OC) and cross-linked-C-telopeptide of type I collagen (CTX) measured in serum (**A**) and saliva (**B**). The *p* values are from Linear regression analysis.

**Figure 3:**
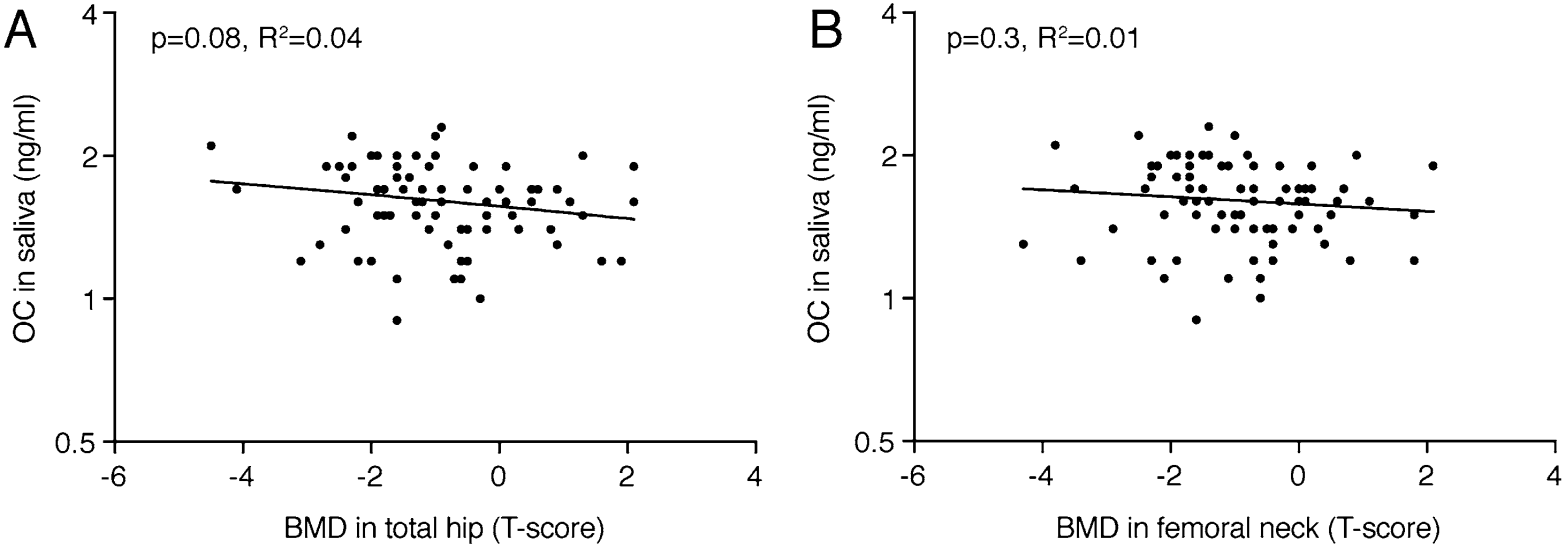
Correlations of osteocalcin determined in saliva with total hip and femoral neck bone mineral density. The data represent 76 data points with bone mineral density (BMD) measured in the (**A**) total hip and (**B**) femoral neck expressed as T-score and bone turnover markers osteocalcin (OC) in saliva. The *p* values are from Linear regression analysis.

**Figure 4:**
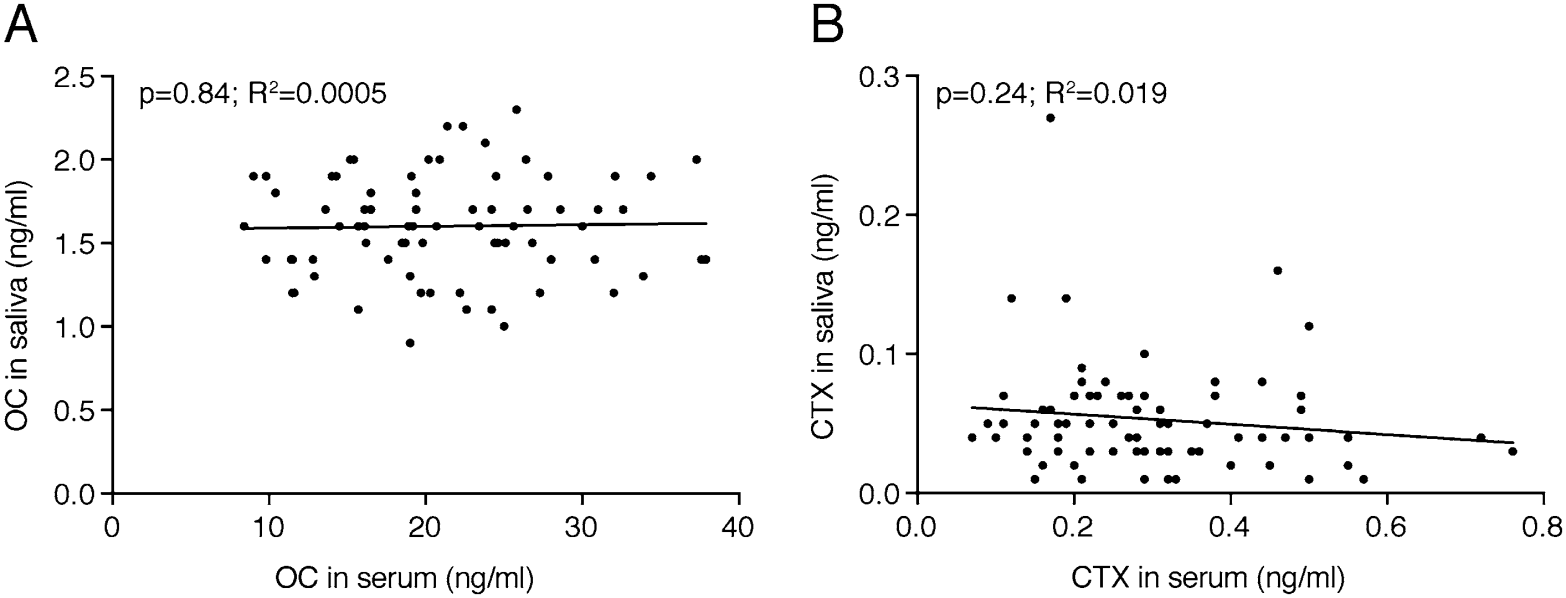
Correlation of bone turnover markers in serum and saliva. The data represent 76 data points from postmenopausal osteoporotic and healthy non-osteoporotic females with bone turnover markers (**A**) osteocalcin (ng/ml) and (**B**) cross-linked-C-telopeptide of type I collagen (CTX ng/ml) measured in the serum and in saliva. The *p* values are from Linear regression analysis.

Spearman correlation analysis, as depicted in Table 2, revealed a strong correlation of serum OC (all < 0.0001) and, to a lesser extent also CTX (*p* = 0.03; *p* = 0.03) values with bone mineral density in lumbar spine and total hip, respectively. There was no correlation of bone mineral density with the respective bone turnover markers in saliva for OC (all *p* > 0.05) and CTX (all *p* > 0.38). However, there was a trend that saliva OC correlates with femoral neck (*p* = 0.16) or total hip (*p* = 0.059) BMD. Linear regression analysis confirms the association of serum OC (R^2^ = 0.15; *p* < 0.0001) and CTX (R^2^ = 0.08; *p* = 0.015) with lumbar spine BMD (Fig. 2A). No association of saliva OC (*p* = 0.8) and CTX (*p* = 0.5) with lumbar spine bone mineral density was observed (Fig. 2B). Linear regression analysis confirms a weak association of saliva OC with total hip BMD (R^2^ = 0.04; *p* = 0.08) and femoral neck BMD (R^2^ = 0.01; *p* = 0.3) (Fig. 3). There was also no association of serum OC (*p* = 0.84) and CTX (*p* = 0.24) with saliva OC and CTX (Fig. 4).

**Table 2:** Spearman correlation between biochemical parameters and bone mineral density of cumulative data.

	Spine aBMD	Femoral neck aBMD	Total hip aBMD	OC	CTX	OC-Sal	CTX-Sal
Spine aBMD	1	0.65**	0.71**	− 0.40**	− 0.24*	− 0.06	− 0.07
Femoral neck aBMD		1	0.91**	− 0.46**	− 0.18	− 0.16	− 0.09
Total hip aBMD			1	− 0.48**	− 0.25*	− 0.22	− 0.10
OC				1	0.69***	0.02	− 0.20
CTX					1	0.08	− 0.17
OC-Sal						1	0.14
CTX-Sal							1

*Oc* osteocalcin, *P1NP* N-terminal propeptide of type I collagen, *CTX* cross-linked-C-telopeptide of type I collagen, *aBMD* areal bone mineral density. Sal is saliva, if not indicated serum.

*p < 0.05; **p < 0.001; ***p < 0.0001.

## Discussion

This research is part of a widespread current effort to unleash the diagnostic potential of saliva as a readily, cheaply and painlessly available source of biological information for a wide range of medical purposes^17^. The main finding of the present study is that current bone turnover markers in serum but not in saliva correlate with bone mineral density in postmenopausal osteoporotic women and healthy non-osteoporotic females. Therefore, stimulated saliva does not appear suitable to replace venipuncture and serum preparation for the measurement of these bone turnover markers.

Several preclinical and clinical studies previously addressed the feasibility of bone turnover markers in saliva. A preclinical rat study investigated potential correlations between saliva and serum markers including CTX^11^. In contrast to our observations, there was a strong correlation of the salivary and the serum CTX levels in sham-operated animals (SHAM) and ovariectomized (OVX) rats. Salivation was stimulated by intraperitoneal injection of pilocarpine. In agreement with our findings, CTX levels were approximately tenfold lower in the saliva compared to the serum. In sheep, serum and saliva levels were in the same range, and OVX sheep showed higher OC levels than SHAM sheep in serum and saliva^10^. In our study, saliva OC levels were tenfold lower than serum levels and only serum—but not saliva—OC correlated with bone mineral density. Thus, preclinical models to some extent are in line with the data we have gained from this cross-sectional human study.

In a previous clinical study on postmenopausal and premenopausal women, saliva and serum bone turnover markers were compared but bone mineral density was not measured^9^. Moreover, serum—as well as saliva—CTX levels were significantly higher than the ones we have measured. No correlation of serum—and saliva-CTX values were performed. Since serum—and saliva-levels, in contrast to our findings, were in the same range, data cannot be compared. The authors of another study with 37 women concluded that osteocalcin levels in saliva correlate with bone mineral density findings^8^, which is in contrast to our study. Osteocalcin serum levels were not reported. Also, there osteocalcin saliva levels were 5 to 10-fold higher than in our saliva group^8^. Thus, there is an obvious difference between the ELISA and the electrochemiluminescence immunoassays, maybe related to salivary mucus interfering with the photometric detection.

The use of saliva as a diagnostic material for systemic conditions relies to a large extent upon the proposition that some plasma components diffuse into the saliva and that this process is not entirely random. The simplest conceptualization envisions some degree of ultrafiltration of the blood plasma into saliva. According to this model, the filtration rate of a certain plasma molecule would largely depend on its size and charge. The degree of correlation between plasma and saliva concentrations would, of course, depend on an entire range of further parameters like, for instance, the stability of the molecule in saliva and the relationship between filtration rate and the volume of saliva produced. Indeed, we know from the vast body of literature on salivary steroid analytics that saliva measurements tend to be tinted with a higher biological variability than serum/plasma measurements. Nevertheless, salivary cortisol is highly correlated with serum free cortisol and the salivary cortisol measurement is meanwhile a widely established and validated diagnostic procedure in many research areas and in the routine clinical practice^18^. However, cortisol is a highly lipophilic small molecule of 362 Da molecular weight that is unlikely to be prematurely degraded in saliva. In contrast, the bone turnover markers analyzed here are much larger, much more polar and much fewer stable peptides/proteins. Still, a look at their molecular weights reveals it is the markers with the lowest molecular weights, CTX being an octapeptide produced by cathepsin K during physiological bone resorption and has MW of only ~ 900 Da^19^ and OC with 5.8 kDa, that we were able to reliably measure in saliva. P1NP with 35 kDa^20^ was only partially and the even larger BAP with 140 kDa^21^ was not at all detectable, although the individual assays have comparable sensitivities in relation to the respective concentration ranges in serum. In addition, the smallest molecule studied, OC also showed correlation trends with some BMD parameters. This really seems to suggest the presence of some filtration-like mechanism at the blood–saliva barrier and allows us to speculate that some still to be discovered even smaller bone turnover marker molecule may finally still render the assessment of the bone turnover in saliva possible.

This is to our knowledge the most comprehensive clinical study of bone turnover markers in saliva to date. Our cohort included representative numbers of both postmenopausal osteoporotic and healthy non-osteoporotic women. We analyzed the currently most widely used bone turnover markers, OC, CTX, P1NP and BAP in saliva and serum, validated the OC and CTX ECLIA measurements for saliva and investigated correlations of these markers in serum and saliva, as well as with bone mineral density. Nevertheless, several limitations also need to be acknowledged. Firstly, it is not an intervention study and we can therefore not rule out that the strong effects of antiresorptive therapies or even anabolic therapies, both affecting bone turnover and causing a significant shift of the bone turnover markers we have used, can be detected and therefore monitored in the saliva. Secondly, only one method of saliva collection was employed. We chose a well-established stimulation protocol, because it appeared most appropriate to yield sufficient saliva in our elderly population of interest. The impact of the saliva collection technique seems to be different for different analytes^8,22–37^. It is therefore possible that other sample collection protocols may be more appropriate for the bone turnover markers studied. Thirdly, we collected only one saliva sample per study participant. Averaging concentrations from several independent samples would definitely improve data robustness. However, we found relatively low intraindividual fluctuations of salivary CTX and especially OC, so that biological variability is unlikely to have roughly falsified our results.

## Conclusion

Our study of the clinically widely used bone turnover markers osteocalcin, cross-linked C-telopeptide of type I collagen, procollagen type I N-terminal propeptide and bone alkaline phosphatase revealed that the smallest molecules, osteocalcin and cross-linked-C-telopeptide of type I collagen can be reliably determined in stimulated saliva by current electroluminescence sandwich immunoassays. However, their salivary concentrations failed to correlate with the respective serum concentrations or bone mineral density in a cohort of postmenopausal osteoporotic and healthy non-osteoporotic females. Their applicability for the salivary assessment of bone metabolism appears therefore unlikely. Novel biomarkers may be needed to pave the way towards a more convenient, non-invasive monitoring of osteoporosis therapies and maybe, one day, even a screening test.
